# The performance of the Mini-Cog in a sample of low educational level elderly

**DOI:** 10.1590/S1980-57642009DN30200003

**Published:** 2009

**Authors:** Sergio Telles Ribeiro Filho, Roberto Alves Lourenço

**Affiliations:** 1 MD, Laboratório de Pesquisa em Envelhecimento Humano, GeronLab, Universidade do Estado do Rio de Janeiro, Rio de Janeiro, RJ, Brazil.; 2 MD, MPH, PhD, Laboratório de Pesquisa em Envelhecimento Humano, GeronLab, Universidade do Estado do Rio de Janeiro, Rio de Janeiro, RJ, Brazil. Internal Medicine Department, Faculdade de Ciências Médicas, Universidade do Estado do Rio de Janeiro, Rio de Janeiro, RJ, Brazil.

**Keywords:** neuropsychology, dementia, mass screening, aging, ambulatory care, validity

## Abstract

**Objectives:**

To study the criterion validity of the Mini-Cog in low educational level elderly.

**Design**

Cross-sectional and validation design.

**Setting**

Policlínica Piquet Carneiro, an outpatient unit of Rio de Janeiro State University Hospital, in Brazil.

**Participants**

A convenient sample consisting of 306 individuals, 65 yrs or older, selected from April 8^th^ to July 15^th^, 2002.

**Methods:**

All participants underwent comprehensive geriatric evaluations which included the Mini-Mental State Examination (MMSE) and the cognitive part of the Cambridge Examination for Mental Disorders of the Elderly – Revised (CAMCOG-R). They were classified as demented or non-demented (DSM-IV). A post-hoc analysis was performed on the data from the 3 word recall test of the MMSE, and the Clock Drawing Test from the CAMCOG-R, and respective scores were added and interpreted in accordance with the Mini-Cog protocol.

**Results:**

293 individuals completed all the study steps; 211 had 4 or less years of schooling and were included in the data analysis. 32% had dementia. Mini-Cog sensitivity and specificity was consistently low independently of the different cut-off points considered. The best performance was found at the cut-off point of 2/3 which yielded sensitivity and specificity of 60% and 65%, respectively.

**Conclusion:**

The Mini-Cog is not a good cognitive screening tool for individuals with less than five years of formal education.

## Abstract (pt)

Estudar a validade de critério do Mini-Cog em idosos com baixo nível educacional.

**Desenho:**

transversal e de validação.

**Cenário:**

Policlínica Piquet Carneiro, uma unidade ambulatorial do Hospital da Universidade do Estado do Rio de Janeiro, no Brasil.

**Participantes:**

Uma amostra de conveniência constituída de 306 indivíduos, 65 anos ou mais, selecionados entre 8 de abril e 15 de julho de 2002.

**Métodos:**

Todos foram submetidos a uma avaliação geriátrica abrangente, que incluiu o Mini-Exame do Estado Mental (MEEM) e a parte cognitiva do Cambridge Examination for Mental Disorders of the Elderly – Revised (CAMCOG-R), e foram classificados segundo sua condição de ser ou não portador de demência (DSM-IV). Foi feita uma análise post-hoc dos dados do teste de evocação de três palavras, incluído no MEEM, e do Teste do Desenho do Relógio, incluído no CAMCOG-R. Os resultados de cada teste foram agrupados e interpretados de acordo com o protocolo do Mini-Cog.

**Resultados:**

293 indivíduos completaram todas as etapas do estudo, e 211 tinham quatro ou menos anos de escolaridade e tiveram seus dados analisados; 32% tinham demência. A sensibilidade e a especificidade do Mini-Cog foram consistentemente baixas, independente do ponto de corte considerado. O melhor desempenho, no ponto de corte 2/3, registrou sensibilidade e especificidade de 60% e 65%, respectivamente.

**Conclusão:**

O Mini-Cog não é uma boa ferramenta para triagem cognitiva de indivíduos com menos de cinco anos de educação formal.

Dementia is common in the geriatric population. It has been suggested that early detection leads to benefits for the patient and their family,^1,2^ and cuts costs.^3^ Early diagnosis thus constitutes an important factor where achieving this depends upon adequate screening performed at the primary care level. However, studies have shown that dementia is underdiagnosed by generalists, and this seems to be due to a relative reluctance of these professionals to apply cognitive screening tests, either because they are time consuming to apply, or because they are perceived as being uncomfortable for the patient or their family.^4-6^

The solution to this problem lies in the development of brief and easily applicable tests that are acceptable to patients, their caregivers, and health professionals. Additionally, these instruments must perform well in populations with heterogeneous characteristics with regard to age, cultural diversity, or diverse levels of education.

This latter point is of particular relevance in developing countries, which tend to have a large proportion of elderly with less than 5 years of formal schooling. More specifically for Brazil, the projected rapid expansion of the elderly population as a whole^7^ will result in a steep increase in the number of patients with dementia. Most of the instruments for detecting dementia currently in use however, such as the Mini-Mental State Examination (MMSE) 8 and the Clock Drawing Test (CDT),^9-14^ were developed in countries where mean educational levels are much higher. Both tests have been shown to perform poorly when applied to very low educational level elderly.^15,16^

The developers of the Mini-Cog sought to enhance CDT performance by adding a simple learning test, namely, the 3 word recall test.^17-20^ In this test, subjects are given a list of three words, just as in the MMSE, and the clock drawing is used as a distracter.

In the original paper, the Mini-Cog performed better than either the MMSE or the CDT alone in a sample with a relatively large ethnic and educational heterogeneity and which had a high prevalence of dementia. However, the number of subjects with less than 5 years’ education was probably low, since the mean educational level of the demented patients was more than 8 years. No separate results for test performances in the subgroup with less than 5 years’ schooling were cited.^17^ In another article, in which the Mini-Cog is applied to a large community-derived sample with a much lower prevalence of dementia, having less than 6 years of formal education was one of the exclusion criteria.^18^ Therefore, there is still little information on the psychometric characteristics of this test in low educational level elderly.

The objective of this study was to determine the accuracy of the Mini-Cog in an elderly population with predominantly low educational levels, treated in a primary care outpatient clinic.

## Method

### Sample selection

Between April 8^th^ and July 15^th^, 2002, a convenient sample of 306 individuals was selected from elderly individuals who were seeking general medical treatment at the Internal Medicine Clinic of the Policlínica Piquet Carneiro, an outpatient unit of Rio de Janeiro State University Hospital. Individuals older than 65 years who attended our screening center seeking a general physician’s office were invited to participate in a study to validate instruments to screen for dementia.^21^ The number of participants recruited daily depended on how many accepted the invitation, and was limited by the absorptive capacity of the research team at the time.

Inclusion criteria were having an age over 65 years and preserved hearing and comprehension, at least enough to fully participate in the study and sign an informed consent form. Exclusion criteria were reports – personal or through an informant – of a serious uncorrected visual or auditory deficiency; being at an advanced stage of cognitive disturbance, or having any mental illness that could compromise understanding and performance on the test procedures; having a native language other than Portuguese; difficulty in hand movement due to rheumatic or neurological diseases. After signing the informed consent form, the subjects were referred for a comprehensive geriatric evaluation, administered by a multi-professional team – a geriatrician, a registered nurse practitioner, a social worker, and a neuropsychologist. At the end of the evaluation, a meeting between the geriatrician and the neuropsychologist classified the patients into one of two dementia syndrome-based groups: demented and non-demented. Therefore, the confirmatory standard for the diagnosis of dementia was the consensual opinion of both professionals, which took into account both the clinical impression and the neuropsychological evaluation, and was based on the DSM-IV^22^ diagnostic criteria for dementia syndrome. Dementia severity was graded in accordance with the modified protocol used by the Brazilian Health Ministry to determine dispensing of medications for patients with Alzheimer’s disease (http://dtr2001.saude.gov.br/sas/PORTARIAS/PORT2002/PT-843.htm). Thus, for the purposes of the present study, those whose scores were less than eight were classified as having severe dementia, those with scores between eight and seventeen points were assigned as having moderate dementia, and those who scored more than seventeen points were classified as having mild dementia. The patients were not further classified as to the cause of their dementia.

The Rio de Janeiro State University Hospital Ethics Committee approved the research protocol, including the Informed Consent Form. The study was supported entirely by the Rio de Janeiro State University.

### Post-Hoc Mini-Cog and study procedures

In addition to the clinical assessment algorithms pertaining to each of the specialties involved, the individuals were submitted to a functional evaluation, which included the Activities of Daily Living Scale^23^ and the Instrumental Activities of Daily Living Scale;^24^ and were submitted to the Geriatric Depression Scale;^25^ and to the MMSE.^8^ The neuropsychometric tests included the MMSE and the cognitive part of the Cambridge Examination for Mental Disorders of the Elderly - Revised (CAMDEX-R),^26^ the CAMCOG-R. This includes a clock drawing task that uses the following instruction: “Draw the face of a large clock, place all the numbers inside and place the hands to show 11:10 (ten minutes past eleven)”. The clock drawing of each patient – copied and identified with its respective register number – had previously been analyzed and scored retrospectively as part of another study,^15^ in accordance with Sunderland’s method, by researchers who had no access to the patient’s file and were blinded to the cognitive condition of the subjects assessed. *Post-hoc* analysis was performed as follows: the clock scores obtained following application of Sunderland’s method greater than or equal to six were considered “normal”, whereas scores less than six were considered “abnormal”. In accordance with the original Mini-Cog methodology, as defined by Borson and Scanlan^17,19^ “The optimal algorithm had the following three rules: subjects recalling none of the words were classified as demented; those recalling all three words were classified as non-demented; and those with intermediate word recall (1–2) were classified based on the CDT (“abnormal=demented; normal=non-demented)”.^17^

The data were entered and analyzed using the program SPSS v 9.0. ROC curves were plotted and the areas under the curves, their confidence intervals, and the best trade-offs between sensitivity and specificity were calculated. Comparisons between categorical variables were made using Pearson’s chi-square test.

## Results

**Figure 1:**
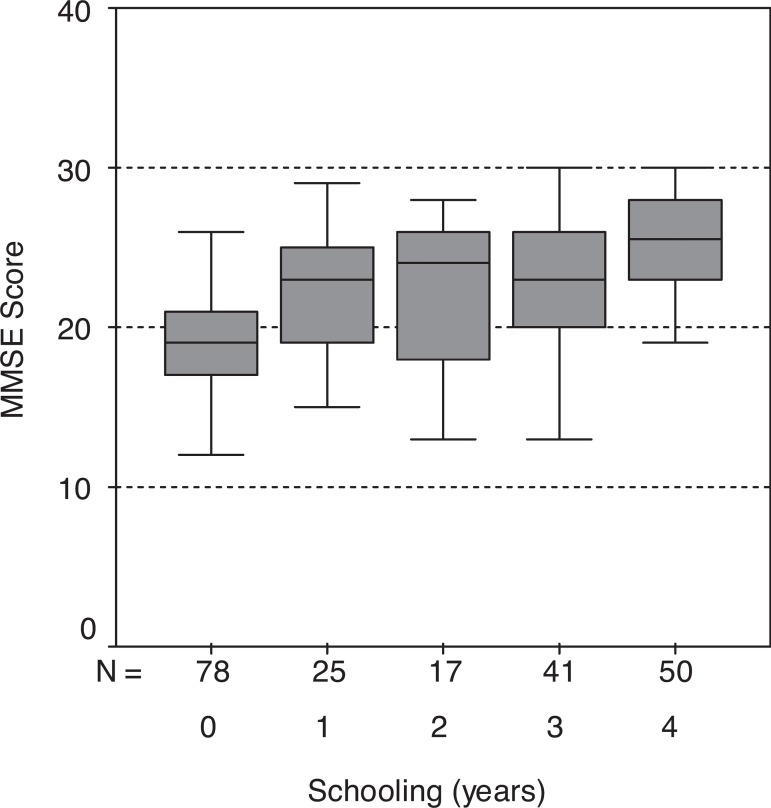
Box plot of Mini-Mental State Examination performance in a sample of low educational level elderly stratified by years of schooling.

A total of 306 subjects were recruited; 293 completed all the study procedures; 211 had 4 or less years of schooling and had their data analyzed. Of these, 153 (72.5%) were female, 59.7% stated they did not live with a partner, 82.5% were not working, 64.9% were retired, and 37% stated they had never attended school. In addition, 71.1% were under 75 years and 12.3% were older than 80 years of age (Table 1). According to the DSM-IV,^22^ 32.2% fulfilled criteria for dementia syndrome. Age ranged from between 65 and 93 years, and had a mean (±SD), median and mode of 72.8 (±5.4), 72, and 73, respectively; mean ages were 74.0 yrs (±5.8) and 72.0 yrs (±5.0) for demented and non-demented groups, respectively. The mean (±SD), median and mode number for years of schooling were 1.8 (±1.7), 2, and 0 respectively. The means (±SD) and median for demented and non-demented groups were 1.28 (±1.49) and 0.5, and 2.06 (±1.66) and 2, respectively. As shown in Table 1, there was a statistically significant difference between demented and non-demented groups in the variables “Work” and “Schooling.” The mean±SD of the MMSE scores for the demented and non-demented groups were 18.8±4.2and 23.3±4.1, respectively (p=0.000), while medians were 19 and 24, respectively. Figure 1 summarizes the performances on the MMSE, stratified by years of schooling.

**Table 1:** Socio-Economic Characteristics Stratified by Dementia Diagnosis - DSM-IV (21) (n= 211) in sample of low educational level elderly.

Items		Dementia (n/%)		Total	p-value
		Yes	No		
Gender	Male	16/27.6	42/72.4	58/27.5	
	Female	52/34.0	101/66.0	153/72.5	0.374
Age (yrs)	65-69	15/24.6	46/75.4	61/28.9	
	70-74	29/32.6	60/67.4	89/42.2	
	75-79	11/31.4	24/68.6	35/16.6	
	≥80	13/50.0	13/50.0	26/12.3	0.144
Schooling (yrs)	0	34/43.6	44/56.4	78/37.0	
	1	8/32.0	17/68.0	25/11.8	
	2	6/35.3	11/64.7	17/8.1	
	3	13/31.7	28/68.3	41/19.4	
	4	7/14.0	43/86.0	50/23.7	0.001
Marital status	Single	6/30.0	14/70.0	20/9.5	
	Separated	8/32.0	17/68.0	25/11.8	
	Widow	28/34.6	53/65.4	81/38.4	
	Married	24/30.4	55/69.6	79/37.4	0.946
Work	Yes	3/10.0	27/90.0	30/14.2	
	No	63/36.2	111/63.8	174/82.5	0.004
Retired	Yes	42/30.7	95/69.3	137/64.96	
	No	25/36.2	44/63.8	9/32.7	0.420

DSM-IV, Diagnostic and Statistical Manual of Mental Disorders, 4th Edition.

Among the 68 individuals classified as having dementia, 44 (65%) were at a mild stage, and 24 (35%) were at a moderate stage whereas no patients had severe dementia.

**Figure 2:**
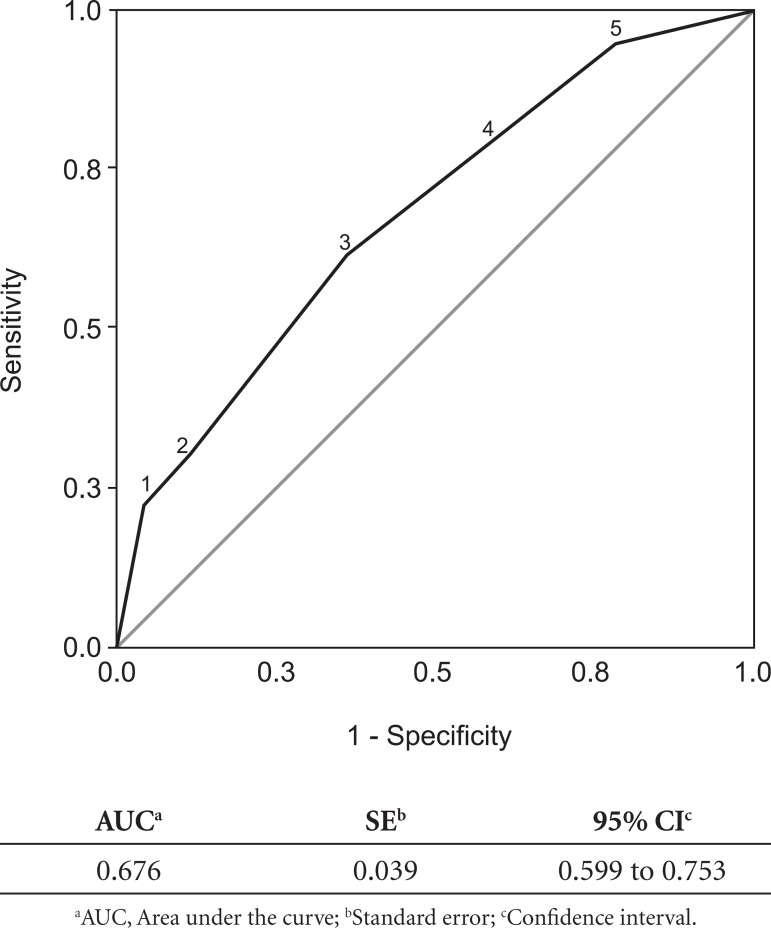
ROC curve of Mini-Cog performance in sample of low educational level elderly.

**Figure 3:**
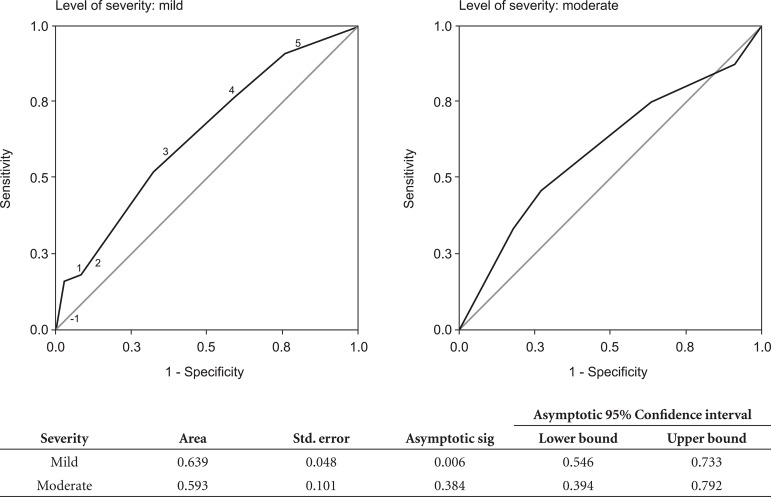
ROC curve of Mini-Cog performance in sample of low educational level elderly, stratified by level of severity.

The sensitivity and specificity of the Mini-Cog were 60% and 65%, respectively, using the original cut-off point of 2/3. Table 2 and Figure 2 demonstrate that using other cut-off points did not improve performance. The accuracy of the Mini-Cog also does not improve when its psychometric characteristics are calculated stratifying the sample by level of dementia severity, as shown in Figure 3.

**Table 2:** Sensitivity and specificity of the MiniCog for different cut-off points in sample of low educational level elderly.

Cut-off points	Sensitivity	Specificity
0/1	0.221	0.958
1/2	0.279	0.902
2/3	0.603	0.650
3/4	0.779	0.427
4/5	0.941	0.224

## Discussion

The accuracy of the Mini-Cog as a screening tool for dementia in this sample of elderly with low educational levels was disappointingly poor. The psychometric characteristics calculated, e.g., sensitivity, specificity, and area under the ROC curve, approached random levels.

This contrasts to its performance in samples with higher levels of education, where it attains sensitivities and specificities greater than 90%^17^. This divergence was not surprising. The hypothesis that education is a crucial variable affecting the results of neuropsychological tests in general was previously demonstrated by Ostroski-Solis et al.^27^ This effect is much stronger than age, for example, and is most significant in those groups with the lowest levels of schooling, especially in those with less than five years formal education. Ardila et al.^28^ compared the performance of illiterates and educated professionals on a wide variety of neuropsychological tests and found that almost all of the abilities tested were strongly influenced by education. Abilities most highly dependent upon on schooling were visual and constructional abilities, abstract reasoning, and memory, including recall of word lists.

We have recently published results which indicate that even the CDT, which was once thought to be resistant to educational heterogeneity, does not in fact perform well in this population group.^15^ Other authors have also described the adverse effect of low schooling levels on CDT performance.^16,29^ The skills needed to draw a clock are apparently highly dependent on formal education, since it is not uncommon for low educational level non-demented subjects to be unable to draw a clock face, yet be able to tell the time indicated on a clock and to reason using concepts linked to the notion of the passage of time,^30^ abilities which are clearly necessary for functional independence. For comparative purposes, application of the CDT alone using Sunderland’s scoring method in our group of patients yielded 59% sensitivity and 64% specificity.^15^

With regard to the 3-item recall test, which is also widely used as a brief test of verbal memory, and has been shown to have a high correlation with the results of the full MMSE,^31,32^ there is less information about how educational levels affect its performance. Even though it has some degree of correlation with other, more sophisticated psychometric tests, there is great variability in results when it is applied to normal individuals, with many non-demented patients recalling zero or only one word.^33,34^ Given Ostroski et al have demonstrated that a six-word recall test was highly influenced by education,^27^ it is likely that the 3 word recall is also influenced by this variable.

Therefore, although the Mini-Cog has definite advantages when applied to more highly educated patient groups it is not surprising that our data does not support its use in older individuals with low levels of education.^35^

Our study had some limitations. One such limitation was that complete information was not available from third parties (caregivers, relatives) for most of the patients evaluated, and this could have led to some misclassification errors.

Another point is that both the clock drawing and 3-word evocation tests were part of the neuropsychological test battery included in our diagnostic criteria algorithm. This information was included as part of that used to establish the diagnosis, and although these items represented only a small section of the full test they may have introduced some information bias.

The third aspect is related to limitations inherent to the post-hoc methodology itself. For example, we used a different scoring system for the clock drawings than that used in the original Mini-Cog. On the other hand, we demonstrated in a previous study that the psychometric properties of four different CDT scoring methods (one of which was Sunderland’s, the others being Shulman’s, Manos’, and Wolf-Klein’s methods) were very similar (they all performed very poorly) in a population of low educational level elderly,^15^ therefore the choice of which to use is probably irrelevant.

On the other hand, a positive aspect was that this sample was composed of individuals who were seeking primary health care, having signs and symptoms not necessarily related to cognitive disorders, and who accepted our invitation over consecutives days during the study period. This probably reduced the problem of selection bias, frequently seen in studies that select their samples based on diagnosis condition of the subjects, with the resultant risk of inflating the accuracy of the test.

In conclusion, the Mini-Cog is strongly influenced by educational level, and although there is some evidence in the international literature that it can be used to screen older subjects with higher educational levels, it appears unsuitable for dementia screening in individuals with less than 5 years of formal education.
